# Urinary Proteome Differences in Patients with Type 2 Diabetes Pre and Post Liraglutide Treatment

**DOI:** 10.3390/cimb45020092

**Published:** 2023-02-06

**Authors:** Mohamed Rafiullah, Hicham Benabdelkamel, Afshan Masood, Aishah A. Ekhzaimy, Mohthash Musambil, Salini Scaria Joy, Assim A. Alfadda

**Affiliations:** 1 Strategic Center for Diabetes Research, College of Medicine, King Saud University, Riyadh 12211, Saudi Arabia; 2 Proteomics Resource Unit, Obesity Research Center, College of Medicine, King Saud University, Riyadh 12211, Saudi Arabia; 3 Department of Medicine, College of Medicine, King Saud University, Riyadh 12211, Saudi Arabia

**Keywords:** GLP-1 receptors agonist, liraglutide, metallothionein-2, proteomics, type 2 diabetes mellitus, zinc-alpha-2-glycoprotein

## Abstract

Diabetes mellitus is a chronic multisystem disease with a high global prevalence. The glucagon-like peptide-1 (GLP-1) receptor agonist liraglutide is known to lower glucose levels and reduce weight. However, the mechanisms underlying the benefits of liraglutide treatment in patients with type 2 diabetes mellitus (T2DM) remain unclear. Twelve male patients with T2DM (pre and post liraglutide treatment) and HbA1c between 8% and 11% were recruited. In the present study, a two-dimensional difference gel electrophoresis (2D-DIGE) matrix-assisted laser desorption/ionization-time of flight (MALDI TOF) mass spectrometric approach combined with bioinformatics and network pathway analysis was used to explore the urine proteomic profile. The mean age of the patients was 52.4 ± 7.5 years. After treatment with liraglutide, a statistically significant change (*p* < 0.006) was observed in HbA1c with no significant changes in body weight or markers of dyslipidemia. Two-dimensional difference gel electrophoresis identified significant changes (≥1.5-fold change, ANOVA, *p* ≤ 0.05) in 32 proteins (4 down- and 28 upregulated) in liraglutide post treatment compared to the pre-treatment state. Albumin, serotransferrin, metallothionein-2 (MT-2), and keratins K1 and K10 were found to be upregulated after liraglutide treatment. The patients showed significant improvement in glycemic control after the 12-week treatment with liraglutide. The renoprotective effect of liraglutide may be linked to the increased urinary abundance of MT-2 and the decreased abundance of zinc alpha 2-glycoprotein (ZAG) and Alpha-1 antitrypsin (α1-AT). More studies are needed to elucidate the molecular mechanisms behind the renoprotective effects of liraglutide.

## 1. Introduction

Diabetes mellitus has become one of the major healthcare problems of modern times. Its prevalence has increased tremendously to reach pandemic levels globally. The number of people with diabetes is expected to increase from 537 million in 2021 to 783 million in 2045 [1]. It was the ninth leading cause of death in 2019 [2]. Uncontrolled diabetes leads to sustained hyperglycemia which damages blood vessels, nerves, and other tissues, causing various diabetic complications. High blood glucose levels in diabetes have been associated with increased mortality due to complications [3]. Hyperglycemia can be controlled by lifestyle modifications and pharmacological treatment. Incretin-based therapy has gained importance in the pharmacotherapy of diabetes in recent years. These treatments improve postprandial glucose levels by enhancing meal-induced insulin secretion [4]. Incretin therapy works by either inhibiting the metabolism of endogenous GLP-1 or acting on the GLP-1 receptors. Patients with type 2 diabetes were shown to have preserved the insulinotropic property of GLP-1, even in those who had unsuccessful sulfonylurea treatment [5].

Liraglutide, a long-acting GLP-1 receptor agonist (GLP-1RA), has been used to treat diabetes and obesity. It increases insulin secretion and inhibits glucagon secretion by acting on the GLP-1 receptor [6]. Liraglutide is 97% homologous to the human GLP-1 and has a plasma half-life of 13 h [7]. As a GLP-1RA, the pharmacological effects of liraglutide mimic those of GLP-1. In addition to its effect on controlling blood glucose and body weight [8], liraglutide was found to decrease cardiovascular deaths, non-fatal myocardial infarction, and non-fatal stroke in people with type 2 diabetes [9]. The cardiovascular benefits of liraglutide may be partly attributed to its effect on weight loss, decreasing blood pressure, and improving the lipid profile [10]. Liraglutide treatment reduced immune-mediated inflammatory responses by decreasing the expression of cytokines such as TNF-α, IL-1β, and IL-6 in patients with type 2 diabetes regardless of weight loss or glycemic control [11]. A meta-analysis of 18 randomized controlled trials found that liraglutide treatment improved renal outcome indicators such as albumin-to-creatinine ratio, serum creatinine, and cystatin C [12]. In animal studies, attenuation of oxidative stress and inflammation in the renal tissues was found to play a key role in the renoprotective action of liraglutide [13]. Several animal and in vitro studies have investigated the molecular mechanisms of the different pharmacological effects of GLP-1RAs [14]. However, clinical studies are limited and most have relied on the analysis of targeted biomarkers. To study the molecular changes behind the liraglutide effects, an untargeted proteomic analysis would be more appropriate.

Proteomic analysis provides a global picture of overall changes in the abundance of proteins in the body. Given the complexity of liraglutide action, involving multiple pathways and organs, an untargeted proteomic approach would be better suited to investigate its molecular mechanisms. Our previous study on the post-liraglutide plasma proteomics of type 2 diabetes patients revealed decreased levels of acute-phase proteins and an improved cardio-metabolic profile [15]. Liraglutide treatment led to protein changes in the renal tissues of diabetic animals. It inhibited the expression of TNF-α and NF-κB in the podocytes of diabetic mice [16]. Proteomic analysis of the renal tissues of liraglutide-treated diabetic mice showed an increased abundance of antioxidant enzymes including glutathione peroxidase-3 and catalase [17]. Changes in the protein abundance in the blood as well as in the kidney tissues are likely to be reflected in the urine proteome. Assessment of urinary inflammatory markers after diabetes treatment has previously been reported [18]. Moreover, urine is a potential source of non-invasive biological fluid that can yield the identification of protein biomarkers. This study evaluated urinary proteins in patients with diabetes who were treated with liraglutide using an untargeted proteomic approach and identified altered protein pathways using bioinformatics and network pathway analysis.

## 2. Materials and Methods

### 2.1. Ethical Considerations and Informed Consent

All of the study procedures were performed in accordance with the ethical standards of the Declaration of Helsinki and the International Conference on Harmonization Good Clinical Practice guidelines. The Institutional Review Board, College of Medicine, King Saud University Hospital, approved the study protocol (no. E-18-3075). Written informed consent was obtained from all of the participants.

### 2.2. Study Subjects

Twelve male patients with T2DM who were followed up in the endocrine outpatient clinic at King Saud University Medical City (KSUMC) with a history of uncontrolled diabetes (HbA1c between 8% and 11%) were recruited. Patients with indications of add-on liraglutide were started on treatment by their physician in a scaled-up dose from 0.6 mg to 1.8 mg of a once-daily subcutaneous injection over a period of three weeks. The follow-up visit was scheduled 3 months after receiving the full dose (1.8 mg) of liraglutide. Urine samples were collected at two time points: one sample before and another sample after treatment with liraglutide. Blood samples were collected by venipuncture into plain tubes (Vacutainer, BD Biosciences, San Jose, CA, USA) from each patient after a 10 h fast. The plasma was separated by centrifugation (15 min, 3000× *g*), divided into several aliquots, and stored at −80 °C for further analysis. The primary endpoint was a reduction in baseline HbA1c of ≥0.5%. The sample size was determined by carrying out a power analysis using the Progenesis SameSpots non-linear dynamics statistical software (version 3.3, Nonlinear Dynamics Ltd., Newcastle, UK) for the determination of the minimum number of required biological replicates, as shown in Figure S1.

### 2.3. Urine Collection and Protein Extraction

Midstream spot urine samples (50–100 mL) were obtained from the participants after a standard 10 h of fasting into a sterile urine container and transported immediately on ice to prevent microbe contamination and proteolysis. Urine analysis was carried out to determine the presence of urinary protein, infection, sugar, or occult blood using a urine test strip (Combur10 Test, Roche, Basel, Switzerland). The samples were then processed and insoluble materials were removed by centrifugation at (4000 rpm) at 4 °C for 10 min, within 30 min of collection, to prevent protein release from these artifacts. The supernatant (5 mL) was carefully removed, aliquoted, and stored at −80 °C for long-term storage. Proteins were isolated from the urine samples, as described previously [19]. The protein pellets were solubilized in labeling buffer (7 M urea, 2 M thiourea, 30 mM Tris−HCl, and 4% CHAPS, pH 8.5). Insoluble material was pelleted by centrifugation (12,000× *g*, room temperature (RT) 15 min), and protein concentrations were determined in triplicate using the 2D-Quant kit (GE Healthcare, Chicago, IL, USA).

### 2.4. Protein Labeling with Cyanine Dyes

Fifty µg of proteins from each sample (12 pre and 12 post) were labeled on ice for 30 min in the dark with 400 pmol of CyDyes (CyDyeTM DIGE Fluor dyes, GE Healthcare, Buckinghamshire, UK) according to the manufacturer’s instructions and run in 12 different gels. In each gel, two samples were analyzed from two different groups. Each sample was covalently labeled with a fluorophore, either Cy3 or Cy5. An equal quantity of all of the samples was pooled, labeled with Cy2, and utilized as an internal standard, which was standardized and matched across gels to eliminate gel-to-gel variance. To prevent dye-specific bias, a dye-switching technique was used during labeling. The distribution of samples in each gel is described in Table S1.

### 2.5. 2D-DIGE and Image Scanning

One-dimensional analytical gel electrophoresis was performed, followed by two-dimensional sodium dodecyl sulfate polyacrylamide gel electrophoresis (SDS-PAGE) on 12.5% fixed concentration gels, as previously described [20,21,22]. Briefly, 1 mg from the pool was added to the rehydration buffer (7 M urea, 2 M thiourea, 4% CHAPS, 0.006 g DTT, 2 µL bromophenol blue, 5 µL IPG buffer (pI 3–11), a protease inhibitor cocktail) and applied to IPG strips. Isoelectric focusing was performed on an Ettan IPGphor IEF Unit (GE Healthcare, 30 V, 12 h, 20 °C at 50 µA per strip). Furthermore, the strips were treated by equilibration buffer with 0.1 g DDT and 0.9 g Iodoacetamide, respectively. Next, standard SDS-PAGE was preformed using an Ettan DALTsix Vertical System (GE Healthcare). The gels were scanned with a Sapphire biomolecular imager (Azure Biosystems, Dublin, OH, USA) and digitalized with Sapphire capture system image analysis software (Azure Biosystems, Dublin, OH, USA).

### 2.6. Colloidal Coomassie Blue Staining of the Preparative Gel

For the preparative gel, total protein (1 mg) was obtained from a pool of equal amounts of protein from the 24 samples. Then, the the protein samples were separated by first and second dimensions with the same conditions in the DIGE section. The gels were fixed in 40% (*v*/*v*) ethanol containing 10% acetic acid overnight and then washed (3 X, 30 min each, ddH2O). The gels were incubated (1 h, 34% (*v*/*v*) CH_3_OH containing 17% (*w*/*v*) ammonium sulfate and 3% (*v*/*v*) phosphoric acid) prior to the addition of 0.5 g/L Coomassie G-250. After 5 days, the stained gels were briefly rinsed with Milli-Q water and stored until the spots were picked and identified by mass spectrometry. Later, the Coomassie blue-stained gel spots were excised manually into a 96-well plate [20,21,22].

### 2.7. Statistical Analysis

The laboratory values’ data are presented as the mean standard deviation. The statistical significance of the difference between the biochemical values of the two groups was analyzed by a paired Student’s *t*-test, with a value of *p* < 0.05 considered as significant. In terms of statistical analyses for gel image analysis, the 2D-DIGE gel images were uploaded to the Progenesis SameSpots software (Nonlinear Dynamics, Newcastle, UK) and analyzed using an automated spot detection method. Although automatic analysis was performed to detect all of the spots across all 24 gel images, each selected spot was verified and manually analyzed wherever necessary. Normalized volumes were used to identify the differentially expressed spots. A cut-off ratio of ≥1.5 fold was considered significant.

### 2.8. Protein Digestion and MALDI Analysis

The Coomassie blue-stained gel spots were washed and digested, as previously described [20,21]. Briefly, destaining was carried out using 50 mM NH_4_HCO_3_ and 50% CH_3_CN, then by adding 100% CH_3_CN. The gel pieces were dried, rehydrated, and digested by adding 15 µL of 20 µg ice-cold trypsin solution in 25 mM NH_4_HCO_3_, 5 mL CH_3_CN, and 5 mL distilled water and incubated for 20 min at 4 °C. Digestion continued overnight at 37 °C. To stop the reduction and extraction the peptides, 1 μL of 1% trifluoroacetic acid was added to the gel pieces and placed in a vortex incubator (1 h, 400 rpm, 25 °C). Finally, a mixture of tryptic peptides (0.8 μL) derived from each protein was spotted onto a MALDI target (384 MTP Anchorchip) (800 μm Anchorchip; Bruker Daltonics, Bremen, Germany). The spectra were obtained using an UltraflexTerm time-of-flight (TOF) mass spectrometer equipped with an IR-laser device (Bruker Daltonics, Bremen, Germany) at reflector and detector voltages of 21 kV and 17 kV, respectively, as described previously [20,21,22]. The PMFs were calibrated against a standard peptide calibration standard II (Bruker Daltonics, Bremen, Germany). The PMFs were assessed using Flex Analysis software (version 2.4, Bruker Daltonics, Bremen, Germany). The MS data were interpreted using BioTools v3.2 (Bruker Daltonics, Bremen, Germany). The peptide masses were searched against the Mascot search algorithm (v2.0.04, updated on 9 May 2021; Matrix Science Ltd., London, UK). The identified proteins were screened for Mascot scores higher than 56 and *p* < 0.05.

### 2.9. Bioinformatics Analysis

The PPI network of differentially expressed proteins was constructed through the STRING database (https://string-db.org/, accessed on 12 October 2022) and was used to analyze protein interaction networks and the functions of the urine proteins differentially expressed in liraglutide pre-treated and post-treated samples. The STRING database allows UniProt IDs into the ingenuity knowledge base, the largest manually curated resource combining information from all published scientific studies. This software aids in determining the functions and pathways that are most strongly associated with the MS-generated protein list by overlaying the experimental expression data onto networks constructed from published interactions. The identified proteins were additionally classified into different categories according to their function and location using the PANTHER classification system (http://www.pantherdb.org, accessed on 30 October 2022).

## 3. Results

### 3.1. Clinical and Biochemical Data

The baseline characteristics of patients are shown in Table 1. The study participants were 52.4 ± 7.5 years of age (mean). In comparison to the pre-treatment data, we did not find any significant changes in body weight, BMI, renal function markers, or markers of dyslipidemia after liraglutide treatment. The HbA1c levels showed a statistically significant change after liraglutide treatment (pre: 9.6% ± 1.2; post: 8.3 ± 1.7). Considering the short period of the treatment (3 months), a change in HbA1c of 1.1% after taking liraglutide was considered clinically significant.

### 3.2. Proteomic Analysis and Identification of Differentially Expressed Proteins

**Figure 1:**
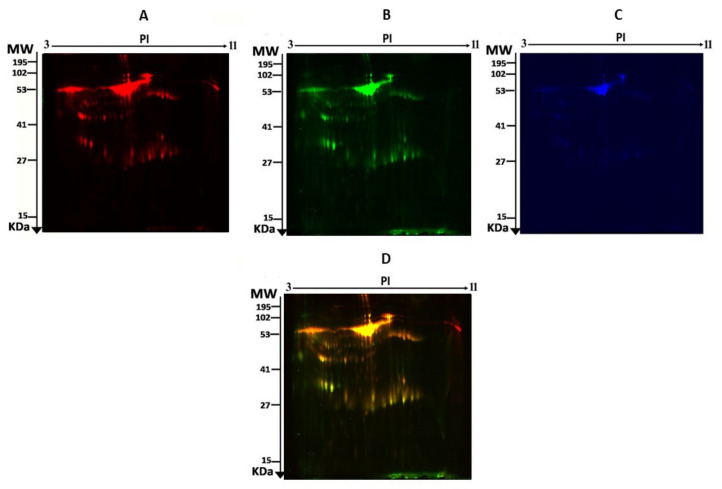
Representative fluorescent protein profile of a two-dimensional difference in gel electrophoresis (2D-DIGE) containing urine sample from post-treatment with liraglutide labeled with Cy3 (**A**), pre-treatment labeled with Cy5 (**B**), a pooled internal control labeled with Cy2 (**C**), and a merged image of PRE/POST (**D**).

**Figure 2:**
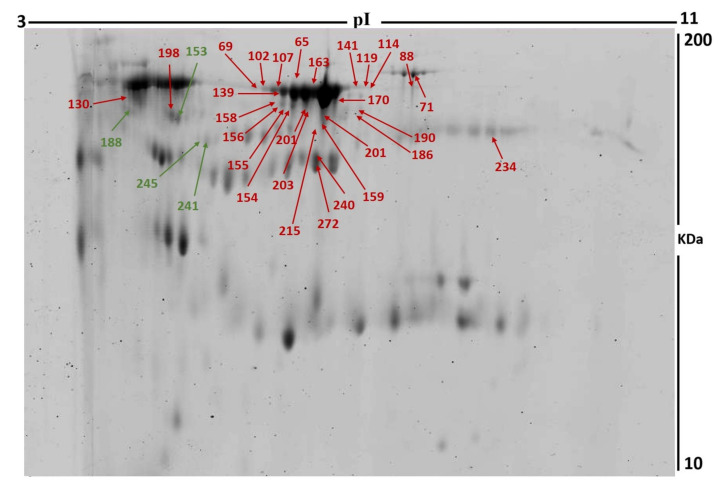
2D-DIGE numbered spots indicate proteins with differential abundance (defined as fold-change ≥ 1.5, *p* ≤ 0.05) between pre-treated and post-treated samples successfully identified with matrix-assisted laser desorption/ionization time-of-flight (MALDI TOF) mass spectrometry (MS). MW, protein molecular weight; pI, isoelectric point.

To assess the differential protein expression among liraglutide-treated individuals (24 samples from 12 gels), we performed 2D-DIGE and MALDI-TOF MS. Figure 1 shows the representative fluorescent protein profiles of a 2D-DIGE of post-treated samples labeled with Cy3 (Figure 1A), pre-treated samples labeled with Cy5 (Figure 1B), a pooled internal control labeled with Cy2 (Figure 1C), and merged 2D-DIGE gels of samples labeled with Cy3/Cy5 (Figure 1D). A total of 980 spots were detected matching across all 24 gel images. Figure S2 shows a total of 58 spots statistically significant on the gels, (ANOVA, *p* ≤ 0.05; fold-change ≥ 1.5) between the pre-treatment and post-treatment samples. Among these, 32 were successfully identified, Figure 2. The spot patterns were reproducible across all 24 gel images, leading to alignment and further analysis. Normalization across the complete set of gels and quantitative differential analysis of the protein levels were achieved using an internal standard with Cy2 labeling. The 32 spots showing a statistical significance between the two conditions were then manually excised from the preparative gel for protein identification by MS.

Peptide mass fingerprints (PMFs) successfully identified 32 of the 58 protein spots excised from the preparative gel. MALDI-TOF mass spectrometry identified 18 spots as unique protein sequences that were matched to entries in the SWISS-PROT database by Mascot with high confidence scores (Table 2, Supplementary Table S2). The sequence coverage of the proteins identified by PMF ranged from 9% to 68%. In a few cases, variants of the same protein were found at several locations on the gel (Table 2, Figure 2). Of the 32 proteins identified, 28 protein spots were upregulated and 4 were downregulated following treatment with liraglutide (Table 2, Figure 2). The significantly upregulated proteins included keratin, type I cytoskeletal 10 (2.7-fold, *p* = 0.022), zinc finger protein 354C (2.7-fold, *p* = 0.009), albumin (2.8-fold, *p* = 0.05), serotransferrin (2.4-fold, *p* = 0.019), and AT-rich interactive domain-containing protein 5B (2.4-fold, *p* = 0.003). A complete list is provided in Table 2. The significantly downregulated proteins included coiled-coil domain-containing protein 13 (*p* = 0.009) and zinc-alpha-2-glycoprotein (*p* = 0.005); a full list is provided in Table 2. Among the identified proteins, zinc-alpha-2-glycoprotein, albumin, cytoplasmic dynein 1 heavy chain 1, keratin, type I cytoskeletal 10, serotransferrin, and zinc-alpha-2-glycoprotein were found in more than one spot on the gels, which could be associated with their post-translational modifications, cleavage by enzymes, or the presence of different protein species (Table 2).

### 3.3. Principal Component Analysis

**Figure 3:**
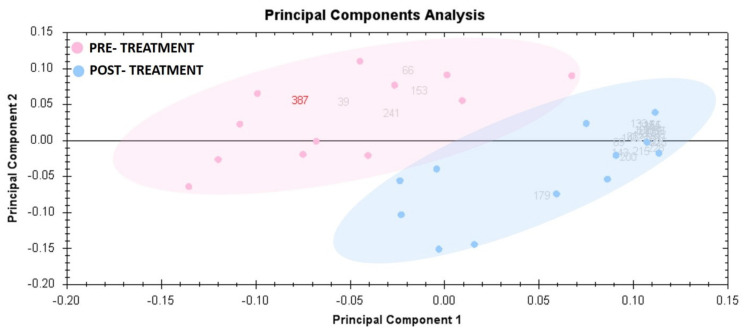
Principal component analysis of the proteomic dataset. Pink dots denote the 12 liraglutide pre-treated urine samples, and blue dots indicate the 12 liraglutide post-treated samples. Together, these explained 60.95% of the selected spots’ variability values. Colored dots and numbers represent gels and spots, respectively.

PCA was used to visualize each study group and detect outliers. Figure 3 displays the score plots for the two study groups. The samples were given group-specific colors. The PCA model demonstrating that the pre-treatment and post-treatment groups clustered in a two-dimensional score plot indicated that the proteomics profile was significantly different between the two groups. In Figure 3, the primary source of variance (PC1, explaining 60.95% of the variance) allows for separation of the pre-treatment group (red) and post-treatment group (blue).

### 3.4. Protein–Protein Interaction (PPI) Network Construction

**Figure 4:**
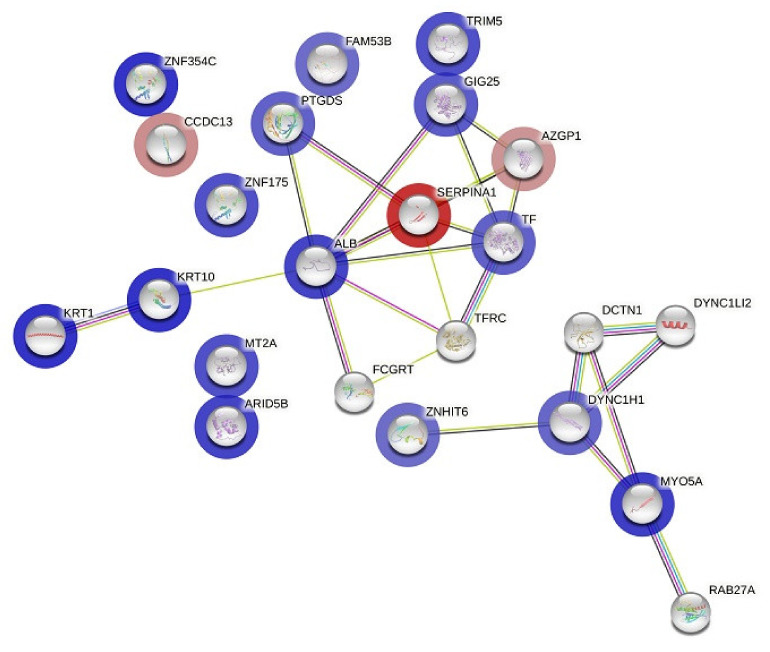
The differentially expressed proteins’ most enriched interaction network in both the pre- and post-treatment phases. Protein nodes with a blue or red halo denote upregulation or downregulation, respectively. Protein nodes without a halo are proposed by the STRING database and indicate potential targets that were functionally coordinated with the differentially expressed proteins. The solid black line displays co-expression; the green line indicates the gene neighborhood; the dark-blue line indicates gene co-occurrence; the purple line shows experimentally determined protein interactions. Interaction network of differentially expressed proteins in the pretreatment group compared to the post-treatment group.

We searched for known and predicted interactions for the differentially expressed proteins identified by 2D-DIGE proteomics in the STRING protein–protein interaction database and constructed a protein–protein interaction network (Figure 4). We identified two networks, with 23 nodes and 25 edges. The network predicted an interaction between alpha-1-antitrypsin (SERPIN A1) and zinc-alpha-2-glycoprotein (ZAG), identified as being downregulated in the liraglutide post-treatment group, and serotransferrin and albumin, which were increased in our study. Biological process pathways identified in STRING for the 32 proteins included gene sets involved with vesicle-mediated transport (FDR < 0.00007), membrane trafficking (FDR < 0.00038), platelet degranulation (FDR < 0.0111), and the immune system (FDR < 0.0132). A second network predicted by STRING involved dynein chain proteins (DYNC1) and was involved in vasopressin-regulated water reabsorption (FDR < 0.007) (Table S3).

**Figure 5:**
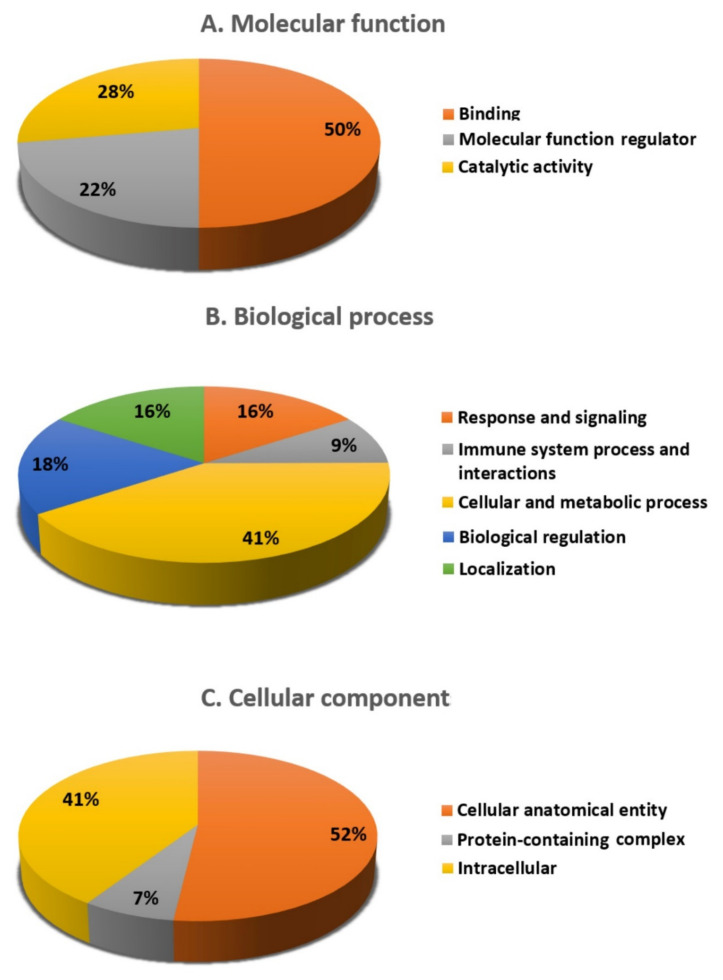
Comparative representation (%) of reported proteins divided into groups based on their cellular components (**A**), molecular functions (**B**), and biological processes (**C**).

The protein analysis through an evolutionary relationships (PANTHER) system was used for the classification of identified proteins according to their molecular functions (Figure 5A), biological processes (Figure 5B), and cellular components (Figure 5C). The functional category showed that most of the differentially expressed proteins identified were enzymes with binding (50%) followed by catalytic activity (28%) (Figure 5A). With regard to biological processes, the majority of the identified proteins were involved in cellular and metabolic processes (41%), followed by biological regulation (18%) (Figure 5B). The majority of the identified proteins were located in the cellular anatomical entity (52%), followed by the intracellular region (41%) (Figure 5C).

## 4. Discussion

This study has described the changes in the abundance of urinary proteins of patients with type 2 diabetes who received 12 weeks of liraglutide treatment. Patients showed significant improvement in glycemic control, though the other parameters, such as body weight, lipid profile, eGFR, and albumin-to-creatinine ratio, did not change. Liraglutide is known to decrease body weight in patients with diabetes. Most studies have reported the liraglutide effect after 26 weeks of treatment [23]. The shorter duration and lower dosage used in this study might not have been sufficient to show a significant reduction in body weight. Yin et al. showed that three months of treatment with liraglutide did not decrease body weight in patients with type 2 diabetes [24]. Similarly, long-term liraglutide treatment was needed to detect significant changes in the lipid profile. Even high-dose liraglutide treatment for 12 weeks did not produce significant changes in the lipid profile compared to a placebo [25]. The lipid profile of our study participants was already within the normal range in the baseline. Therefore, a significant change after the liraglutide treatment was not expected. Similarly, the eGFR was also in the normal range in the baseline and did not change significantly after liraglutide treatment. The results of urine proteomic analysis revealed differential expression of 32 proteins. The comparison of protein levels between the post- and pre-treatments identified the upregulation of 28 proteins and the downregulation of 4 proteins.

### 4.1. Proteins Increased after Liraglutide Treatment

Liraglutide treatment led to a significantly increased urinary abundance of keratin type II cytoskeletal 1, zinc finger protein 354C, albumin, serotransferrin, and AT-rich interactive domain-containing protein 5B. It is interesting to note that the abundance of albumin levels increased significantly after treatment. Biochemical data show unremarkable changes in ACR and eGFR between the pre- and post-treatments. In a clinical trial, 26-week treatment with liraglutide did not change the renal function in the LIRA-RENAL trial [26]. It was attributed to the smaller sample size and shorter duration of treatment. An increased abundance of albumin in the urinary proteome with no sign of microalbuminuria has been reported in previous proteomic studies [27,28,29,30]. Therefore, the increase in urinary albumin abundance after treatment is unlikely to reflect the renal outcomes of liraglutide. There was a slight decrease in ACR but it was not statistically significant. Serotransferrin is another protein that was found to be excreted at higher levels after liraglutide treatment. Urinary serotransferrin is an early predictor of diabetic nephropathy. The urinary excretion of serotransferrin is reported to increase with the increase in urinary albumin [31]. Therefore, the increase in urinary serotransferrin excretion is understandable. However, the serum proteomics of these patients who received the liraglutide showed a decrease in their serotransferrin levels [15]. Therefore, the increase in the urinary levels of serotransferrin is likely to have come from the increased glomerular permeability or the decreased tubular resorption [32].

Metallothioneins (MT) are a group of cysteine-rich metal-binding proteins that are expressed in various organ tissues and involved in metal ion homeostasis. MTs are also potent antioxidants and can protect tissues against oxidative-stress-induced injury [33]. Overexpression of MTs in different tissues is reported to protect them from diabetes-induced injury. Renal overexpression of MT prevented renal injury [34] and its deficiency accelerated the damage in the renal tissues of diabetic animals [35]. Therefore, the increased abundance of metallothionein-2 (MT-2) in our study could be an outcome of the possible renoprotective action of the liraglutide. Our previous work on the plasma proteomics of liraglutide-treated patients did not show an increased abundance of MT-2 [15]. Therefore, it is highly likely that MT-2 was overexpressed in the renal cells. The keratins type II cytoskeletal 1 (K1) and type I cytoskeletal 10 (K10) were found to be higher after the liraglutide treatment. Human kidney cells express only four keratins, K7, K8, K18, and K19 [36]. Therefore, the keratins K1 and K10 are likely to have been excreted from the blood. K1 and K10 are known markers of the keratinized epithelium [37]. The relationship between diabetes or the liraglutide treatment and the increased excretion of K1 and K10 is not clear. Overexpression of K1 and K10 has not been reported in people with diabetes to date.

### 4.2. Proteins Decreased Post Liraglutide Treatment

This study has identified a decreased urinary abundance of proteins such as coiled-coil domain-containing protein 13, zinc-alpha-2-glycoprotein, and alpha-1-antitrypsin. Zinc-alpha-2-glycoprotein (ZAG), an adipokine, is expressed mainly in adipocytes and is suggested to regulate adipose mass [38]. In obesity, ZAG is found to be under-expressed. It increases the utilization of lipids by promoting lipolysis in white adipose tissue and increasing the expression of uncoupling protein-1 in brown adipose tissue. ZAG is also reported to contribute to weight loss [39]. It is known to be associated with insulin resistance in individuals with prediabetes and newly diagnosed diabetes [40]. ZAG is suggested to play an important role in regulating insulin signaling and sensitivity in the adipose through its association with insulin receptor substrate-1 and glucose transporter-4 [41]. Urinary ZAG levels are found to be higher in individuals with type 2 diabetes in clinical studies [42]. It has been suggested to be an early biomarker of diabetic nephropathy. Urine ZAG levels were correlated positively with albumin to creatinine ratio (ACR) and negatively with estimated glomerular filtration rate (eGFR) [42]. Patients with micro- and macroalbuminuria were reported to have higher urine ZAG levels [43]. The protective effect of liraglutide on kidney function has previously been reported in humans [12] and animals [44]. Therefore, the decreased urinary abundance of ZAG in the present study can be attributed to the renoprotective action of liraglutide.

Alpha-1-antitrypsin (α1-AT), an acute-phase reactant protein, is expressed in the urinary extravascular vesicles of people with diabetes. It has previously been reported to gradually increase with declining kidney function. α1-AT is shown to be a useful marker in predicting diabetic kidney disease before the onset of microalbuminuria and in the prognosis of diabetic kidney disease [45]. α1-AT is also upregulated by inflammation. Liraglutide ameliorates diabetic nephropathy [44] as well as inflammation [46]. Therefore, the substantially lower levels of α1-AT in our study could be an indication of improvement in kidney function and lowered inflammation in patients treated with liraglutide.

The protein–protein interaction network identified the interaction between the downregulated proteins Zinc-α-2-glycoprotein and alpha-1-antitrypsin and upregulated proteins albumin and serotransferrin. Zinc-α-2-glycoprotein is an adipokine, modulated by liraglutide administration. The liraglutide treatment increased the circulating Zinc-α-2-glycoprotein level and was associated with increased insulin sensitivity [40]. Similarly, the deficiency of alpha-1-antitrypsin has been reported to be associated with type 2 diabetes [47]. In addition, urinary alpha-1-antitrypsin was found to be an early marker of diabetic nephropathy [45]. Liraglutide treatment decreased the urinary excretion of both of these proteins. This will indirectly increase the serum levels of these proteins and is likely to have contributed to the antidiabetic action of liraglutide. Upregulated albumin and serotransferrin are the other two proteins with higher interaction in the pathway analysis. These proteins are usually excreted in an analogous pattern in urine. However, since the abundance of albumin in the urinary proteome can increase without microalbuminuria [27,30], the increased albumin and serotransferrin are unlikely to reflect the renal outcomes of liraglutide treatment.

The present study found that the 12-week treatment with liraglutide treatment led to a differential abundance of proteins that are indicative of improved renal function and decreased inflammation. Even though there was no change in the ACR or eGFR, liraglutide treatment might have been renoprotective, as evidenced by the increased urinary abundance of MT-2 and the decreased abundance of ZAG and α1-AT. It is understandable that the molecular changes that contribute to the improvement in kidney function were activated after the liraglutide treatment. The renoprotective effect of liraglutide may be linked to the differential regulation of proteins MT-2, ZAG, and α1-AT. Further investigation is needed to identify how liraglutide induces these changes to produce a renoprotective effect. Liraglutide treatment also increased the urinary abundance of keratins K1 and K10. To date, these keratin proteins have not been observed in an altered state in people with diabetes. We do not know whether K1 and K10 mediate any pharmacological effect of liraglutide. The strength of this study is that we compared the urine proteomics of patients before and after treatment with liraglutide. A major limitation of this study is that it is difficult to differentiate between increased urinary excretion and upregulated proteins. We used 2D-DIGE and MALDI-TOF techniques to identify the differentially excreted proteins in the urine; this needs to be confirmed using immunoblotting techniques.

## 5. Conclusions

The present study revealed the differences in the urinary abundance of proteins after liraglutide treatment. A 12-week treatment period with liraglutide significantly improved glycemic control and altered the urinary abundance of proteins. Albumin, serotransferrin, MT-2, and keratins K1 and K10 were found to be upregulated and the abundance of ZAG and α1-AT was decreased. The increased urinary abundance of MT-2 and decreased abundance of ZAG and α1-AT may be linked to the renoprotective effect of liraglutide. Larger cohort studies are required to be carried out to explore the molecular mechanisms behind this effect.

## Figures and Tables

**Table 1:** Clinical and biochemical characteristics of the study population before and after liraglutide treatment.

	Pre-Treatment	Post-Treatment	*p*-Value
	Mean ± SD	Mean ± SD	
Height (cm)	155.2 ± 4.5	155.2 ± 4.5	-
Weight (kg)	87 ± 14	85.5 ± 11.5	0.40
BMI (kg/m^2^)	36.2 ± 6.1	36 ± 4.3	0.46
HbA1C (%)	9.6 ± 1.2	8.3 ± 1.7	0.006 *
Total cholesterol (mmol/L)	4.5 ± 1.5	4.4 ± 1.3	0.43
LDL (mmol/L)	2.3 ± 1.4	2.3 ± 1.2	0.47
HDL (mmol/L)	1.3 ± 0.3	1.2 ± 0.5	0.44
TG (mmol/L)	1.9 ± 0.64	1.6 ± 0.42	0.13
Urea (mmol/L)	4.6 ± 2.3	4.4 ± 1.8	0.3
Creatinine (µmol/L)	61.3 ± 17.2	61.7 ± 19.1	0.47
eGFR	93.2 ± 26.7	96.1 ± 24.0	0.38
A/C ratio	17.3 ± 9.8	21.08 ± 18.0	0.3

BMI: body mass index; HbA1C: hemoglobin A1c; HDL: high-density lipoprotein; LDL: low-density lipoprotein; TG: triglyceride; eGFR: estimated glomerular filtration rate. * *p* < 0.005.

**Table 2:** List of proteins identified with changes in abundance between liraglutide pre-treated and post-treated in urine samples. Values for the average ratio between the two states are shown with their corresponding levels of fold changes and one-way ANOVA (*p* < 0.05) using 2D-DIGE. Analysis type: MALDI-TOF; database: SwissProt; taxonomy: Homo sapiens.

Sl No.	Spot No. ^a^	Accession No.	Protein Name	MASCOT ID	*p* Value ^b^ (ANOVA)	Ratio ^c^ POST/PRE	Exp ^d^
1	159	P02768	Albumin	ALBU_HUMAN	0.002	2.7	UP
2	201	P04264	Keratin, type II cytoskeletal 1	K2C1_HUMAN	0.003	2.7	UP
3	215	P02768	Albumin	ALBU_HUMAN	0.003	2.3	UP
4	203	Q14865	AT-rich interactive domain-containing protein 5B	ARI5B_HUMAN	0.003	2.4	UP
5	69	P02768	Albumin	ALBU_HUMAN	0.005	2.4	UP
6	156	P02768	Albumin	ALBU_HUMAN	0.005	2.5	UP
7	241	P25311	Zinc-alpha-2-glycoprotein	ZA2G_HUMAN	0.005	0.48	DOWN
8	158	P02768	Albumin	ALBU_HUMAN	0.006	2.4	UP
9	139	Q86Y25	Zinc finger protein 354C	Z354C_HUMAN	0.009	2.7	UP
10	141	Q9C035	Tripartite motif-containing protein 5	TRMIM5_HUMAN	0.009	2.2	UP
11	153	Q8IYE1	Coiled-coil domain-containing protein 13	CCD13_HUMAN	0.009	0.45	DOWN
12	88	Q9Y4I1	Unconventional myosin-Va	MYO5A_HUMAN	0.009	2.5	UP
13	154	Q9Y473	Zinc finger protein 175	ZN175_HUMAN	0.01	2.2	UP
14	155	P02768	Albumin	ALBU_HUMAN	0.011	2.3	UP
15	227	P02795	Metallothionein-2	MT2_HUMAN	0.016	2.2	UP
16	114	P02787	Serotransferrin	TRFE_HUMAN	0.019	2.4	UP
17	186	P13645	Keratin, type I cytoskeletal 10	K1C10_HUMAN	0.022	2.7	UP
18	163	P02768	Albumin	ALBU_HUMAN	0.023	2.6	UP
19	245	P25311	Zinc-alpha-2-glycoprotein	ZA2G_HUMAN	0.025	0.53	DOWN
20	65	P02768	Albumin	ALBU_HUMAN	0.028	1.9	UP
21	272	Q9NWK9	Box C/D snoRNA protein 1	BCD1_HUMAN	0.028	1.7	UP
22	130	P01011	Alpha-1-antichymotrypsin	AACT_HUMAN	0.037	2	UP
23	71	P02787	Serotransferrin	TRFE_HUMAN	0.037	1.9	UP
24	188	P01009	Alpha-1-antitrypsin	A1AT_HUMAN	0.04	0.19	DOWN
25	119	P02768	Albumin	ALBU_HUMAN	0.04	2.4	UP
26	102	Q14204	Cytoplasmic dynein 1 heavy chain 1	DYHC1_HUMAN	0.043	2.1	UP
27	107	P02768	Albumin	ALBU_HUMAN	0.052	2.8	UP
28	240	P41222	Prostaglandin-H2 D-isomerase	PTGDS_HUMAN	0.053	1.9	UP
29	190	P13645	Keratin, type I cytoskeletal 10	K1C10_HUMAN	0.055	2.7	UP
30	234	Q14204	Cytoplasmic dynein 1 heavy chain 1	DYHC1_HUMAN	0.055	1.8	UP
31	198	Q14153	Protein FAM53B	FA53B_HUMAN	0.055	1.7	UP
32	170	P02768	Albumin	ALBU_HUMAN	0.066	2.4	UP

^a^ Protein accession number for SWISSPROT Database. ^b^ *p* Value (ANOVA). ^c^ Ratio between the groups. ^d^ Protein expression between the groups.

## Data Availability

The datasets used and/or analyzed during the current study are available from the corresponding author upon reasonable request.

## Supplementary Materials

The following supporting information can be downloaded at: https://www.mdpi.com/article/10.3390/cimb45020092/s1: Figure S1: Power calculation; Figure S2: Reference gel with 58 statistically significant spots between the pre-treatment and post-treatment samples (ANOVA, *p* ≤ 0.05; fold-change ≥ 1.5). Table S1: Experimental design; Table S2: Mass spectrometry list of significant differentially abundant proteins; Table S3: Different canonical pathways obtained from STRING database analysis.
