# Supplementary material for: Urinary Proteome Differences in Patients with Type 2 Diabetes Pre and Post Liraglutide Treatment
Source: Curr Issues Mol Biol. 2023 Feb 6;45(2):1407–21. doi: 10.3390/cimb45020092 (PMC9956006; doi:10.3390/cimb45020092)
Supplement: Supplementary file 1 [file cimb-45-00092-s001.zip › cimb-2165285-supplementary.pdf]

Figure S1: Power calculation for determination of the minimum number of required biological variants for 2-DIGE analysis. The power curve was used to calculate the sample size required to find significant difference with a fold-change of ≥1.5 between two paired groups at 86.6% power and p-value ≤ 0.05.

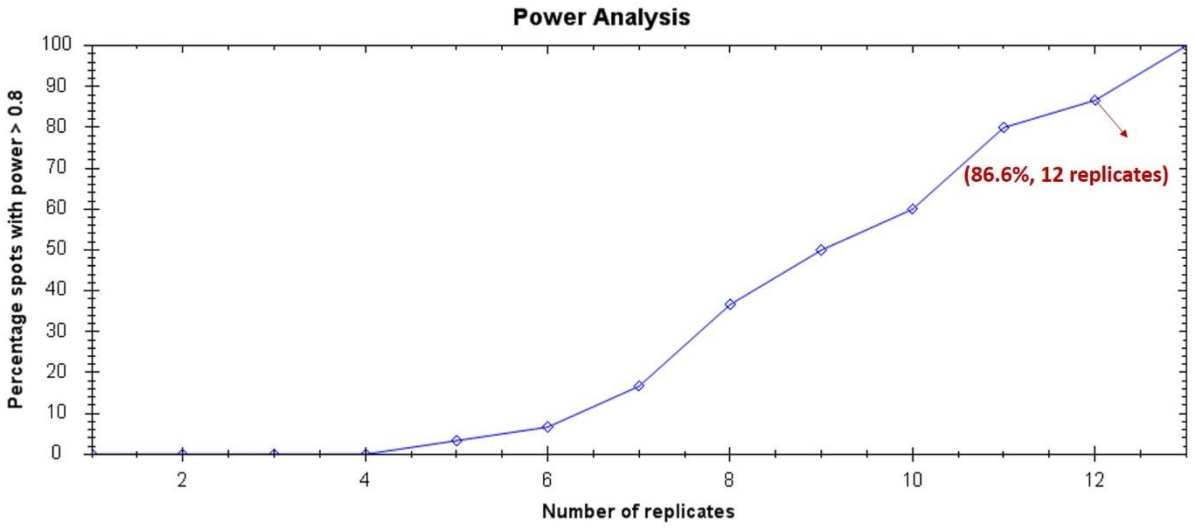

Figure S2: Reference gel with statistically significant 58 spots between the pre-treatment and post-treatment samples (ANOVA, p ≤ 0.05; fold-change ≥ 1.5).

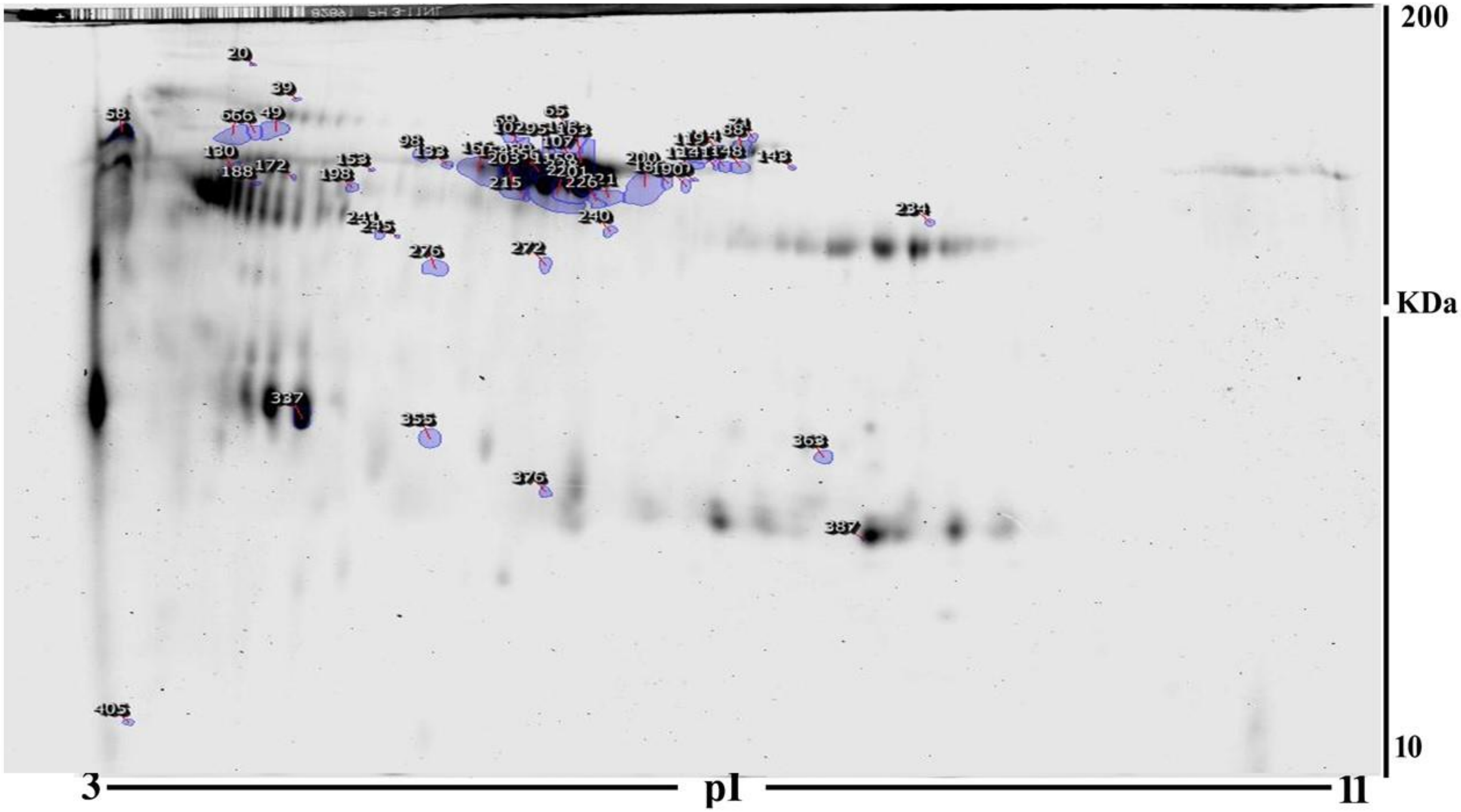

Table S1: Experimental design: 24 samples run on 12 2D-PAGE gels, samples were labeled randomly with Cy3 and Cy5, and a pooled sample was used as an internal standard and was stained with Cy2.

Gel	Cy3	Cy5	Cy2
1	1	P1	Pooled sample
2	P2	2	Pooled sample
3	3	P3	Pooled sample
4	P4	4	Pooled sample
5	5	P5	Pooled sample
6	P6	6	Pooled sample
7	7	P7	Pooled sample
8	P8	8	Pooled sample
9	9	P9	Pooled sample
10	P10	10	Pooled sample
11	11	P11	Pooled sample
12	P12	12	Pooled sample

Table S2: Mass spectrometry list of significant differentially abundant proteins between liraglutide pre-treated and post –treated states identified in urine samples, using 2D-DIGE with. Protein name, accession number, Mascot score, MS % coverage, protein MW and pI values according to Uniprot database are listed. a Protein accession number for SWISSPROT Database.

Sl no:	Spot No[a]	Accession No[b]	Protein Name	MASCOT ID	Pi[c]	MW[d]	Cov%	Score[e]
1	159	P02768	Albumin	ALBU_HUMAN	5.92	71317	50	215
2	201	P04264	Keratin, type II cytoskeletal 1	K2C1_HUMAN	8.15	66170	36	124
3	215	P02768	Albumin	ALBU_HUMAN	5.92	71317	22	72
4	203	Q14865	AT-rich interactive domain-containing protein 5B	ARI5B_HUMAN	8.89	1334339	9	57
5	69	P02768	Albumin	ALBU_HUMAN	5.92	71317	57	162
6	156	P02768	Albumin	ALBU_HUMAN	5.92	71317	33	79
7	241	P25311	Zinc-alpha-2-glycoprotein	ZA2G_HUMAN	5.57	4079	16	59
8	158	P02768	Albumin	ALBU_HUMAN	5.92	71317	23	72
9	139	Q86Y25	Zinc finger protein 354C	Z354C_HUMAN	8.36	66458	18	56
10	141	Q9C035	Tripartite motif-containing protein 5	TRMIM5_HUMAN	5.73	575000	18	56
11	153	Q8IYE1	Coiled-coil domain-containing protein 13	CCD13_HUMAN	8.83	80834	24	56
12	88	Q9Y4I1	Unconventional myosin-Va	MYO5A_HUMAN	8.70	216545	17	56
13	154	Q9Y473	Zinc finger protein 175	ZN175_HUMAN	8.48	84009	39	57
14	155	P02768	Albumin	ALBU_HUMAN	5.92	71317	23	56
15	227	P02795	Metallothionein-2	MT2_HUMAN	8.28	7178	36	56
16	114	P02787	Serotransferrin	TRFE_HUMAN	6.81	79280	31	132
17	186	P13645	Keratin, type I cytoskeletal 10	K1C10_HUMAN	5.13	59020	21	62
18	163	P02768	Albumin	ALBU_HUMAN	5.92	71317	55	219
19	245	P25311	Zinc-alpha-2-glycoprotein	ZA2G_HUMAN	5.57	34079	20	68
20	65	P02768	Albumin	ALBU_HUMAN	5.92	71317	65	181
21	272	Q9NWK9	Box C/D snoRNA protein 1	BCD1_HUMAN	5.62	54511	14	51
22	130	P01011	Alpha-1-antichymotrypsin	AACT_HUMAN	5.33	97792	21	56
23	71	P02787	Serotransferrin	TRFE_HUMAN	6.81	79280	59	240
24	188	P01009	Alpha-1-antitrypsin	A1AT_HUMAN	5.47	46878	56	74
25	119	P02768	Albumin	ALBU_HUMAN	5.92	71317	62	170
26	102	Q14204	Cytoplasmic dynein 1 heavy chain 1	DYHC1_HUMAN	6.01	534809	11	57
27	107	P02768	Albumin	ALBU_HUMAN	5.92	71317	22	62
28	240	P41222	Prostaglandin-H2 D-isomerase	PTGDS_HUMAN	7.66	21243	36	59
29	190	P13645	Keratin, type I cytoskeletal 10	K1C10_HUMAN	5.13	59020	68	68
30	234	Q14204	Cytoplasmic dynein 1 heavy chain 1	DYHC1_HUMAN	6.01	534809	12	60

31	198	Q14153	Protein FAM53B	FA53B_HUMAN	6.69	94756	11	56
32	170	P02768	Albumin	ALBU_HUMAN	5.92	71317	53	126

a Spot number.
b Theoretical isoelectric point.
c Theoretical relative mass.
d MASCOT coverage
e MASCOT score

Table S3: The table shows the different canonical pathways obtained from STRING database analysis.

**KEGG pathways**						
**#term ID**	**term description**	**observed gene count**	**background gene count**	**strength**	**false discovery rate**	**matching proteins in your network (labels)**
hsa04962	Vasopressin-regulated water reabsorption	3	44	2	0.007	DYNC1LI2,DYNC1H1,DCTN1
**REACTOME**						
**#term ID**	**term description**	**observed gene count**	**background gene count**	**strength**	**false discovery rate**	**matching proteins in your network (labels)**
HSA-5653656	Vesicle-mediated transport	9	660	1.06	###	DYNC1LI2,ALB,DYNC1H1,TFRC,DCTN1,RAB27A,MYO5A,TF,SERPINA1
HSA-199991	Membrane Trafficking	8	622	1.04	0	DYNC1LI2,DYNC1H1,TFRC,DCTN1,RAB27A,MYO5A,TF,SERPINA1
HSA-114608	Platelet degranulation	4	127	1.43	0	ALB,GIG25,TF,SERPINA1
HSA-168256	Immune System	10	###	0.64	0	MT2A,KRT1,DYNC1LI2,DYNC1H1,DCTN1,TRIM5,GIG25,RAB27A,MYO5A,SERPINA1
HSA-199977	ER to Golgi Anterograde Transport	4	152	1.35	0	DYNC1LI2,DYNC1H1,DCTN1,SERPINA1
HSA-3371497	HSP90 chaperone cycle for steroid hormone receptors (SHR)	3	54	1.67	0	DYNC1LI2,DYNC1H1,DCTN1
HSA-6811436	COPI-independent Golgi-to-ER retrograde traffic	3	50	1.71	0	DYNC1LI2,DYNC1H1,DCTN1
HSA-6798695	Neutrophil degranulation	5	473	0.95	0	KRT1,DYNC1H1,GIG25,RAB27A,SERPINA1

HSA-6807878	COPI-mediated anterograde transport	3	100	1.41	0	DYNC1LI2,DYNC1H1,DCTN1
HSA-8957275	Post-translational protein phosphorylation	3	107	1.38	0	ALB,TF,SERPINA1
**Diseases**						
**#term ID**	**term description**	**observed gene count**	**background gene count**	**strength**	**false discovery rate**	**matching proteins in your network (labels)**
DOID:9120	Amyloidosis	5	70	1.78	0.0001	KRT1,AZGP1,ALB,GIG25,SERPINA1
DOID:0060158	Acquired metabolic disease	6	320	1.2	0.0035	KRT1,AZGP1,ALB,TFRC,GIG25,SERPINA1
DOID:0050636	Familial visceral amyloidosis	3	21	2.08	0.0041	AZGP1,ALB,SERPINA1
DOID:10611	Protein-losing enteropathy	2	2	2.93	0.0086	ALB,SERPINA1
DOID:0060832	Griscelli syndrome type 1	2	3	2.75	0.0115	RAB27A,MYO5A
DOID:0060877	Bullous congenital ichthyosiform erythroderma	2	3	2.75	0.0115	KRT1,KRT10
DOID:3263	Piebaldism	2	4	2.63	0.0123	RAB27A,MYO5A
DOID:4603	Epidermolytic hyperkeratosis	2	4	2.63	0.0123	KRT1,KRT10
DOID:16	Integumentary system disease	6	534	0.98	0.014	KRT1,KRT10,ALB,RAB27A,MYO5A,SERPINA1
DOID:0050736	Autosomal dominant disease	8	1163	0.77	0.0152	KRT1,KRT10,DYNC1H1,DCTN1,GIG25,RAB27A,MYO5A,SERPINA1
DOID:174	Acanthoma	2	8	2.33	0.0215	KRT1,KRT10

DOID:0014667	Disease of metabolism	7	997	0.78	0.0337	KRT1,AZGP1,ALB,TFRC,TRIM5,GIG25,SERPINA1
DOID:0050739	Autosomal genetic disease	10	2323	0.56	0.0431	FCGRT,KRT1,KRT10,DYNC1H1,DCTN1,TRIM5,GIG25,RAB27A,MYO5A,SERPINA1
DOID:5082	Liver cirrhosis	2	15	2.05	0.0484	ALB,SERPINA1
**Tissue expression**						
#term ID	term description	**observed gene count**	**background gene count**	**strength**	**false discovery rate**	**matching proteins in your network (labels)**
BTO:0000121	Bile	3	19	2.13	0.003	AZGP1,TF,SERPINA1
BTO:0000759	Liver	11	1882	0.7	0.003	FCGRT,MT2A,KRT10,AZGP1,ALB,DYNC1H1,TFRC,DCTN1,GIG25,TF,SERPINA1
BTO:0004850	Bone marrow cell	5	170	1.4	0.003	TFRC,PTGDS,GIG25,TF,SERPINA1
BTO:0000345	Digestive gland	12	2645	0.59	0.0045	FCGRT,MT2A,KRT10,AZGP1,ALB,DYNC1H1,TFRC,DCTN1,PTGDS,GIG25,TF,SERPINA1
BTO:0001491	Viscus	16	5020	0.43	0.0045	FCGRT,MT2A,KRT1,DYNC1LI2,KRT10,ARID5B,AZGP1,ALB,FAM53B,DYNC1H1,TFRC,DCTN1,PTGDS,GIG25,TF,SERPINA1
BTO:0000237	Cerebrospinal fluid	3	45	1.75	0.0056	PTGDS,TF,SERPINA1
BTO:0000392	Plasma cell	4	137	1.4	0.0056	TFRC,PTGDS,GIG25,SERPINA1
BTO:0000545	Gut	3	56	1.66	0.0076	ALB,FAM53B,DYNC1H1
BTO:0001703	Right atrium	3	54	1.67	0.0076	ALB,TF,SERPINA1
BTO:0001702	Left atrium	3	61	1.62	0.0085	ALB,TF,SERPINA1
BTO:0000574	Hematopoietic cell	7	933	0.8	0.0097	KRT1,KRT10,ALB,DYNC1H1,TFRC,DCTN1,SERPINA1
BTO:0000775	Lymphocyte	6	648	0.9	0.0107	KRT1,KRT10,ALB,DYNC1H1,DCTN1,SERPINA1
BTO:0001629	Left ventricle	3	74	1.54	0.0109	ALB,TF,SERPINA1
BTO:0001279	Spinal cord	4	213	1.2	0.011	DYNC1H1,PTGDS,TF,SERPINA1
BTO:0001486	Skeletal system	7	1203	0.69	0.0298	ALB,FAM53B,TFRC,PTGDS,GIG25,TF,SERPINA1
BTO:0000089	Blood	8	1675	0.61	0.035	KRT1,KRT10,AZGP1,ALB,DYNC1H1,TFRC,DCTN1,SERPINA1

BTO:0001419	Urine	2	27	1.8	0.04	ALB,SERPINA1
BTO:0003091	Urogenital system	16	6716	0.31	0.0446	FCGRT,MT2A,DYNC1LI2,KRT10,ARID5B,AZGP1,ALB,FAM53B,DYNC1H1,TFRC,DCTN1,PTGDS,GIG25,RAB27A,TF,SERPINA1
**Subcellular localization**						
**#term ID**	**term description**	**observed gene count**	**background gene count**	**strength**	**false discovery rate**	**matching proteins in your network (labels)**
GOCC:0031983	Vesicle lumen	6	240	1.33	0.00059	ALB,DYNC1H1,GIG25,RAB27A,TF,SERPINA1
GOCC:0072562	Blood microparticle	5	116	1.56	0.00059	KRT1,ALB,TFRC,GIG25,TF
GOCC:0005615	Extracellular space	9	985	0.89	0.00071	KRT1,AZGP1,ALB,TFRC,PTGDS,GIG25,RAB27A,TF,SERPINA1
GOCC:0034774	Secretory granule lumen	5	230	1.27	0.0039	ALB,DYNC1H1,GIG25,RAB27A,SERPINA1
GOCC:0030141	Secretory granule	7	704	0.93	0.0046	KRT1,ALB,DYNC1H1,GIG25,RAB27A,MYO5A,SERPINA1
GOCC:0005856	Cytoskeleton	9	1526	0.7	0.0093	KRT1,DYNC1LI2,ZNF175,KRT10,ALB,CCDC13,DYNC1H1,DCTN1,MYO5A
GOCC:0030286	Dynein complex	3	51	1.7	0.0093	DYNC1LI2,DYNC1H1,DCTN1
GOCC:1990712	HFE-transferrin receptor complex	2	6	2.45	0.0093	TFRC,TF
GOCC:0005576	Extracellular region	10	2035	0.62	0.01	KRT1,AZGP1,ALB,TFRC,DCTN1,PTGDS,GIG25,RAB27A,TF,SERPINA1
GOCC:0070062	Extracellular exosome	5	368	1.06	0.0119	ALB,TFRC,RAB27A,TF,SERPINA1
GOCC:0031093	Platelet alpha granule lumen	3	68	1.57	0.0136	ALB,GIG25,SERPINA1
GOCC:0031410	Cytoplasmic vesicle	9	1709	0.65	0.0136	KRT1,ALB,DYNC1H1,TFRC,GIG25,RAB27A,MYO5A,TF,SERPINA1

GOCC:0030135	Coated vesicle	4	222	1.19	0.0172	TFRC,RAB27A,TF,SERPINA1
GOCC:0097179	Protease inhibitor complex	2	12	2.15	0.0172	ALB,SERPINA1
GOCC:0070288	Ferritin complex	2	15	2.05	0.0194	ALB,TFRC
GOCC:0005868	Cytoplasmic dynein complex	2	19	1.95	0.0274	DYNC1LI2,DYNC1H1
GOCC:0043226	Organelle	20	9848	0.24	0.0274	KRT1,DYNC1LI2,ZNF175,KRT10,ARID5B,ALB,CCDC13,ZNF354C,FAM53B,DYNC1H1,TFRC,DCTN1,ZNHIT6,PTGDS,TRIM5,GIG25,RAB27A,MYO5A,TF,SERPINA1
GOCC:0012505	Endomembrane system	11	3080	0.48	0.0291	KRT1,ALB,DYNC1H1,TFRC,DCTN1,PTGDS,GIG25,RAB27A,MYO5A,TF,SERPINA1
GOCC:0045111	Intermediate filament cytoskeleton	3	116	1.34	0.0303	KRT1,ZNF175,KRT10
GOCC:1990752	Microtubule end	2	22	1.89	0.0303	DYNC1H1,DCTN1
GOCC:0043232	Intracellular non-membrane-bounded organelle	11	3196	0.47	0.0349	KRT1,DYNC1LI2,ZNF175,KRT10,ALB,CCDC13,FAM53B,DYNC1H1,DCTN1,TRIM5,MYO5A
GOCC:0043229	Intracellular organelle	19	9242	0.24	0.0401	KRT1,DYNC1LI2,ZNF175,KRT10,ARID5B,ALB,CCDC13,ZNF354C,FAM53B,DYNC1H1,TFRC,DCTN1,PTGDS,TRIM5,GIG25,RAB27A,MYO5A,TF,SERPINA1
GOCC:0099513	Polymeric cytoskeletal fiber	4	330	1.01	0.0411	KRT1,KRT10,DYNC1H1,DCTN1
